# The side population of ovarian cancer cells defines a heterogeneous compartment exhibiting stem cell characteristics

**DOI:** 10.18632/oncotarget.2053

**Published:** 2014-06-01

**Authors:** Maximilian Boesch, Alain G. Zeimet, Daniel Reimer, Stefan Schmidt, Guenther Gastl, Walther Parson, Franziska Spoeck, Jiri Hatina, Dominik Wolf, Sieghart Sopper

**Affiliations:** ^1^ Internal Medicine V, Innsbruck Medical University, Innsbruck, Austria; ^2^ Tyrolean Cancer Research Institute, Innsbruck, Austria; ^3^ Department of Gynecology and Obstetrics, Innsbruck Medical University, Innsbruck, Austria; ^4^ Institute of Legal Medicine, Innsbruck Medical University, Innsbruck, Austria; ^5^ Department of Biology and Biomedical Centre, Faculty of Medicine Pilsen, Charles University Prague, Pilsen, Czech Republic; ^6^ Medical Clinic III, Oncology, Hematology and Rheumatology, University Clinic Bonn (UKB), Bonn, Germany

**Keywords:** Cancer stem cell, cancer-initiating cell, side population, ABC drug transporter, epithelial ovarian cancer, tumor heterogeneity

## Abstract

Cancer stem cells (CSC) are believed to be involved in tumor evasion of classical antitumor therapies and have thus become an attractive target for further improvement of anticancer strategies. However, the existence and identity of CSC are still a matter of controversy. In a systematic screen of 13 ovarian cancer cell lines we show that cells with stem cell properties are reliably detectable as a minor population, characterized by ABC transporter expression resulting in the side population (SP) phenotype. In different cell lines, either ABCG2 or ABCB1 was found to be responsible for this effect. Purified SP cells featured virtually all characteristics of *bona fide* CSC, including clonogenicity, asymmetric division and high tumorigenicity *in vivo*. Using in-depth phenotyping by multicolor flow cytometry, we found that among the investigated ovarian cancer cell lines the SP compartment exhibits tremendous heterogeneity and is composed of multiple phenotypically distinct subpopulations. Thus, our study confirms previous results showing that CSC are contained within the SP. However, the exact identity of the CSC is still disguised by the high complexity of the CSC-containing compartment. Further functional studies are needed to determine whether a single cellular subset can unambiguously be defined as CSC or whether multiple stem cell-like cells with different properties coexist. Moreover, the observed heterogeneity may reflect a high level of plasticity and likely influences tumor progression, escape from immune-surveillance and development of resistance to anticancer therapies and should therefore be considered in the development of new treatment strategies.

## INTRODUCTION

Cancer stem cells (CSC), or cancer-initiating cells, define a population of cancer cells with unique functional properties. CSC have been implicated in both cancer initiation and metastatic spread during cancer progression [1, 2]. In addition, CSC have been shown to resist the efficacy of cytotoxic and targeted anticancer agents [3-5], potentially leading to disease recurrence. However, the identity of CSC still remains to be defined in detail in many solid tumor entities, hampering progress towards the development of CSC-directed therapeutics.

Ovarian cancer is a tumor entity in which most patients respond favorably to primary treatment, thereby achieving clinical remission [6]. Yet, the majority will experience disease recurrence with concurrent acquisition of drug resistance and fatal outcome [7]. This clinical behavior of ovarian cancer suggests that this malignancy might be a prototypical stem cell-driven tumor type [8].

Although ovarian CSC have been described by several independent research groups, a consensus marker set for this cell population is still lacking. Some groups have reported that ovarian CSC reside in the CD44^high^ fraction [9, 10], whereas others described these cells to be enriched in the aldehyde dehydrogenase (ALDH)^+^/CD133^+^ compartment [11] or that ovarian CSC exhibit a side population (SP) phenotype [12-14]. Similarly, ovarian CSC have also been identified using the surface marker CD24 [15]. These controversial data highlight the need for a systematic screen for ovarian CSC markers using different model systems. This may finally allow the elucidation of a more consistent profile of this clinically highly relevant cell population.

In this study, we comprehensively screened 13 human ovarian cancer cell lines and primary ovarian tumor tissue for the presence of populations with phenotypic and functional properties of CSC. We provide evidence that the SP phenotype, in contrast to most other CSC markers, is a common denominator of cells with functional characteristics of stem cells, including clonogenicity, asymmetric division and tumorigenicity in mouse models. Flow cytometry-based in-depth analysis of SP and non-SP (NSP) fractions finally disclosed a plurality of distinct cell subsets in both cell compartments, demonstrating a very high degree of heterogeneity even in established cell lines. Our results reinforce the SP phenotype as a promising candidate marker for ovarian CSC but also provide evidence for a high degree of phenotypic variability within the putative CSC compartment, a finding with broad implications for biology and therapy of ovarian cancer.

## RESULTS

### SP and ALDH^+^ Subsets are Commonly Found in Ovarian Cancer Cell Lines

**Figure 1:**
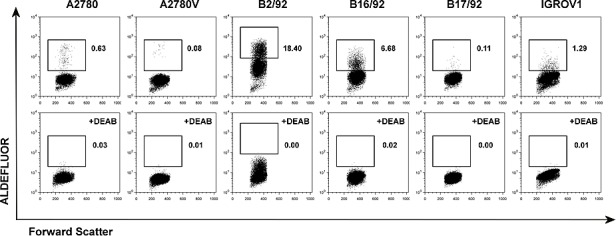
Screening of various ovarian cancer cell lines for ALDH^+^ subsets Cell lines were stained for ALDH enzymatic activity and analyzed by flow cytometry. ALDH^+^ subsets are indicated by rectangular gates, and the percentage of cells within these gates is given (upper row). Corresponding DEAB inhibition controls are shown in the lower row. Data are representative examples of at least three independent experiments. ALDH, aldehyde dehydrogenase; DEAB, diethylaminobenzaldehyde.

**Figure 2:**
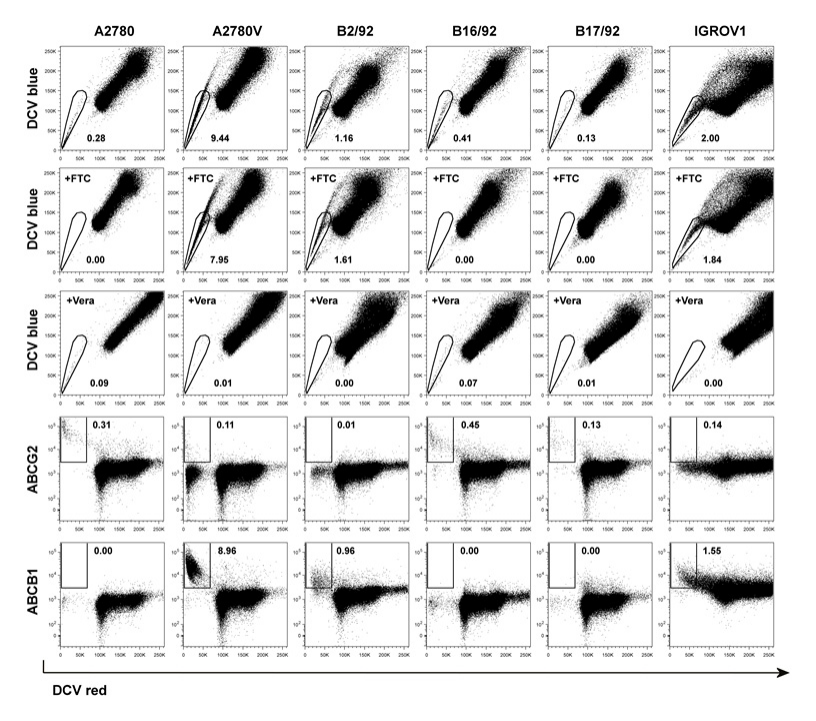
Screening of various ovarian cancer cell lines for SP subsets Cell lines were stained using DCV and analyzed by flow cytometry. SP subsets are indicated by polygonal gates, and the percentage of cells within these gates is given (first row). Corresponding FTC (second row) and verapamil (Vera; third row) inhibition controls are shown. DCV-stained cells were subsequently stained using fluorochrome-conjugated monoclonal antibodies directed against ABC drug transporters. Rectangular gates indicating positive staining for ABCG2 (fourth row) and ABCB1 (fifth row) are shown, and the percentage of cells within these gates is given. Data are representative examples of at least three independent experiments. SP, side population; DCV, Vybrant^®^ DyeCycle^TM^ Violet; FTC, fumitremorgin C.

To characterize the relevance of various CSC markers in ovarian cancer cells, we performed systematic phenotyping of a panel of ovarian cancer cell lines (A2780, A2780V, B2/92, B16/92, B17/92, IGROV1; see Suppl. Table 1 for cell line characteristics) using flow cytometry. Strikingly, the surface markers CD24, CD44, CD90, CD133 and CD326 all failed to consistently identify distinct small subpopulations in the different cell lines. These markers are either not expressed at all or bulk-expressed in many of the cell lines (Suppl. Fig. 1, Suppl. Table 2). In contrast, both ALDH (Fig. 1, Suppl. Table 2) and the SP phenotype (Fig. 2, Suppl. Table 2) robustly identified distinct small subsets (typically ≤2%) in each of the six cell lines. Moreover, distinct small subsets of ALDH^+^ and SP cells could also be detected in 3/7 (Suppl. Fig. 2) and 6/7 (Suppl. Fig. 3) additional ovarian cancer cell lines, respectively (see again Suppl. Table 1 for cell line characteristics). Furthermore in a proof-of-concept study, SP subsets could also be detected in freshly isolated primary ovarian tumor cells (Suppl. Fig. 4A). Of note, the response pattern of SP cells to fumitremorgin C (FTC) (which selectively blocks ABCG2 [16, 17]) and verapamil (which blocks several ABC drug transporters including ABCG2 and ABCB1 [18]) suggested differential drug transporter expression among different SP fractions. In line with these pharmacological drug transporter inhibition data, we found that FTC/verapamil double-sensitive SP (i.e., A2780, B16/92, B17/92) expressed high amounts of ABCG2, whereas verapamil-sensitive and FTC-insensitive SP (i.e., A2780V, B2/92, IGROV1) highly expressed ABCB1 (co-expression of both transporters was not observed) (Fig. 2). Taken together, the presence of distinct minority populations of ALDH^+^ and SP cells, but not of subpopulations defined by other CSC markers, is a common feature of ovarian cancer cell lines (total prevalence: ALDH 9/13 cell lines, SP phenotype 12/13 cell lines).

### SP Cells, But Not ALDH^+^ Cells, Have Consistently Enhanced Single Cell Clonogenicity

**Figure 3:**
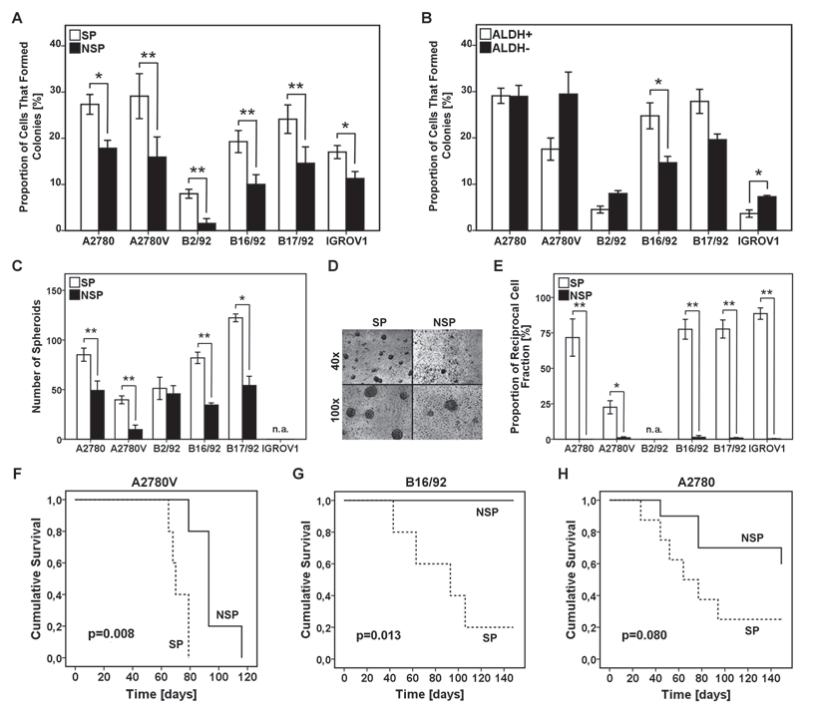
Analysis of SP and ALDH^+^ cells for functional stem cell characteristics (A,B) Analysis of single cell clonogenicity. SP/NSP (A) or ALDH^+^/ALDH^−^ (B) cells were single cell-sorted and subsequently cultured for two weeks. Wells with outgrowing clones were counted and the proportion of cells capable of colony formation was calculated. (C) Analysis of spheroid formation was performed by seeding 1×10^3^ SP or NSP cells on primary mesothelial cell monolayers pre-established in 8-well chamber slides, followed by culturing for one week. The number of spheroids per chamber is depicted. (D) Representative photomicrographs of SP and NSP spheroids at the indicated magnifications (cell line: B17/92). (E) Analysis of asymmetric cell division was performed by single cell sorting of SP or NSP cells. After three weeks of culture, viable clones were analyzed for the SP fraction to determine the reciprocal cell population present in the sample. (F-H) Kaplan-Meier survival curves of female NOD/SCID mice. Mice were intra-peritoneally inoculated with 5×10^4^ SP or NSP cells. Tumor development was regularly monitored and mice with severe tumor burden were euthanized. SP, side population; NSP, non-SP; ALDH, aldehyde dehydrogenase; * p<0.05, ** p<0.01; n.a., not applicable.

To investigate whether ALDH^+^ cells or SP cells (or both) exhibited stem cell traits the clonogenic potential at the single cell level was determined. In all six cell lines tested we found that single cells of the SP had a significantly higher clonogenic potential when compared to their NSP counterparts (Fig. 3A). Conversely, in comparison with ALDH^−^ cells, clonogenicity of ALDH^+^ cells was significantly increased only in one cell line (Fig. 3B). These functional data suggest that SP cells, rather than ALDH^+^ cells, are characterized by an increased colony-forming capacity. This prompted us to focus in more detail on the SP phenotype.

### SP Cells Show Stem Cell Characteristics

We next investigated additional features commonly used to specify *bona fide* CSC, including spheroid formation in a physiologically relevant microenvironment, asymmetric division and tumor engraftment in the NOD/SCID mouse model.

The mesothelial cell layer lining the peritoneal cavity is the primary target site for metastatic tumor cells in advanced-stage ovarian cancer [6]. In order to investigate spheroid formation by SP and NSP cells in this specific microenvironment, we established a co-culture system consisting of primary mesothelial cell monolayers and low numbers (i.e., 1×10^3^) of purified cancer cell fractions. Of five cell lines tested (IGROV1 cells did not form spheroids at all), we observed significantly increased numbers of spheroids in the SP fraction of four models (i.e., A2780, A2780V, B16/92, B17/92), whereas in the fifth cell line (i.e., B2/92) we could only detect a slight trend that did not reach statistical significance (Fig. 3C + 3D). These results demonstrate that SP cells are more efficient than NSP cells in forming spheroids under these physiologically relevant experimental conditions.

We next assessed the ability of SP and NSP cells to produce progeny with unequal fate (i.e., to asymmetrically divide). To this end, clones derived from single cell-sorted cells (either SP or NSP) were analyzed in terms of repopulation of the reciprocal cell population. In all cell lines tested (B2/92 cells could not be sufficiently expanded) asymmetric division was only possible in the SP fraction (Fig. 3E) whereas NSP clone cultures remained SP-negative even after prolonged periods of incubation. These results provide evidence that SP cells, but not NSP cells, can both self-renew and differentiate into a phenotypically different cell type.

To assess the capacity of SP cells to give rise to tumors *in vivo*, we used the NOD/SCID mouse model and chose an orthotopic site of metastasis for engraftment. To this end, 5×10^4^ tumor cells were inoculated into the abdominal cavity of female NOD/SCID mice. In two models (i.e., A2780V, B16/92), mice receiving SP cells succumbed to the tumor burden significantly earlier than did the NSP controls (Fig. 3F + 3G). In further two models (i.e., A2780, IGROV1) similar results were obtained, although the difference in survival curves did not reach statistical significance (Fig. 3H and data not shown). Using B17/92, however, no trend towards superior tumor repopulation by SP cells could be detected (data not shown). Altogether our findings suggest that SP cells are more efficient in propagating tumor growth also under *in vivo* conditions.

Taken together, we have shown that in various ovarian cancer cell lines, SP compartments share the functional properties commonly used to define *bona fide* stem cell populations, suggesting a stem-like nature of ovarian cancer SP cells.

### Multicolor Flow Cytometry Reveals Tremendous Heterogeneity in Ovarian Cancer

**Figure 4:**
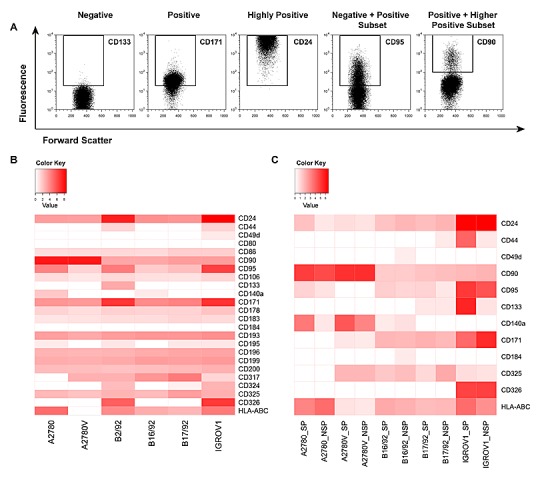
In-depth phenotypic characterization of ovarian cancer cell lines and purified SP/NSP fractions Cells were stained with fluorochrome-conjugated monoclonal antibodies and analyzed by flow cytometry. (A) Illustration of the principal possibilities of marker expression as encompassed in this study. (B) Systematic heatmap analysis of surface marker expression in ovarian cancer cell lines. Shown are MFI above cut-off after normalization to respective controls and log-transformation. (C) Systematic heatmap analysis of surface marker expression in purified SP and NSP fractions. Shown are MFI above cut-off after normalization to respective controls and log-transformation. Data represent the mean of at least two, but typically three, independent experiments. SP, side population; NSP, non-SP; MFI, median fluorescence intensity.

Cells with stem cell properties were enriched but not exclusively found in the SP compartment, and not all SP cells exhibited CSC properties. To further characterize the phenotype and potentially detect a further restricted ovarian CSC identity downstream of the SP denominator [19], we extended the panel to include markers implicated in ovarian cancer progression (e.g., CD140a, CD171) [20, 21], epithelial-to-mesenchymal transition (EMT; e.g., CD325) [22], cell migration/chemotaxis (e.g., chemokine receptors) [23], and interaction with the immune system (e.g., HLA-ABC, CD95) [24, 25] (for complete list see Suppl. Table 3). In these experiments, we observed a broad spectrum of marker expression, ranging from no expression to intermediate and high expression, and expression in distinct subsets (Fig. 4A). More importantly, these analyses showed marked heterogeneity between the various cell lines, with virtually no common pattern in expression levels as determined by median fluorescence intensity (MFI; Fig. 4B). Accordingly, cluster analysis failed to identify marker groups showing relevant clustering (data not shown).

We next sought to comparatively assess the expression of selected markers specifically in the SP and the NSP. To this end, pure SP and NSP fractions were generated and stained for the respective antigens. As an example, HLA-ABC was found to be differentially expressed among the SP/NSP fractions of most cell lines (Fig. 4C, bottom row). Similarly, CD24, CD95, CD140a, CD171 and CD325 were also differently expressed in SP and NSP in the majority of cell lines. Other markers (CD44, CD49d, CD90, CD133, CD184) showed different expression levels between SP and NSP only in a few cell lines and CD326 displayed comparable expression in SP and NSP of all cell lines. Strikingly, with the exception of CD95, which was overrepresented in the SP fraction of essentially all cell lines, virtually none of the markers showed consistent over- or under-expression in the SP or NSP fractions of the investigated cell lines. Accordingly, cluster analysis did not reveal any notable clustering of SP/NSP compartments (data not shown). Together, these data suggest that even the small subsets defined by a conserved CSC marker (i.e., the SP phenotype) exhibit a highly complex marker profile that is unique to the respective population in the individual cell line.

**Figure 5:**
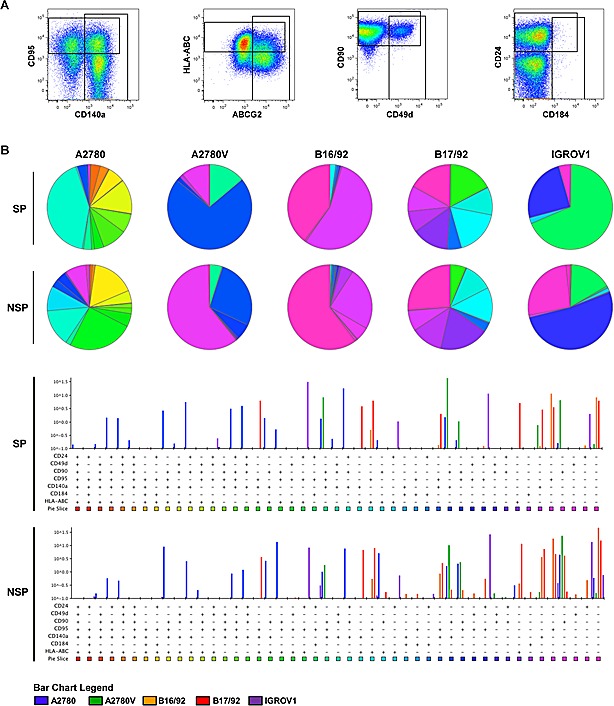
SPICE analysis of ovarian cancer heterogeneity Defined mixtures of corresponding SP and NSP fractions were stained for different markers (staining 1 of Suppl. Table 4) and analyzed by multicolor flow cytometry. (A) Definition of populations positive for individual markers (cell line: A2780). These gates were combined by Boolean operations to obtain proportions of cells within all possible combinations, which were then imported into SPICE for final data analysis. (B) SPICE analysis of ovarian cancer heterogeneity after class-division into SP (upper panel) and NSP (lower panel). Subset distributions are presented in the weighted category mode, and only subsets above a threshold of ≥0.1% are shown. Data are representative examples of at least two independent experiments. SP, side population; NSP, non-SP.

As exemplified in the right two panels of Fig. 4A, several of the markers investigated exhibited biphasic expression, consistent with the presence of distinct subpopulations. Thus, in an attempt to further refine the putative CSC pool, we determined by multicolor flow cytometry whether these markers were co-expressed or expressed on mutually exclusive subpopulations (Fig. 5A). To this end, a combination of eight markers (i.e., SP/NSP discrimination plus seven additionally selected markers, Suppl. Table 4) was used, which theoretically results in 128 distinct cell populations of both SP and NSP. To exclude cell populations with limited biological significance, the cut-off for the definition of a *bona fide* cell population was set to 0.1% of total cells (corresponding to >85 cells). As results for such a vast number of parameters are difficult to visualize using common strategies we used SPICE, a software specifically designed for this purpose (Fig. 5B). The individual subpopulations are color-coded and their relative abundance is reflected by the size of sectors in the pie charts or the height of bars in the bar charts below. By simply comparing the color pattern of the marker combination CD24, CD49d, CD90, CD95, CD140a, CD184 and HLA-ABC, it becomes clear that the difference between cell lines is bigger than that between SP (upper panels) and NSP (lower panel) of the individual cell lines. Moreover, the level of heterogeneity as measured by the number of subsets is different between the cell lines, with A2780 being the most heterogeneous (19 populations above the threshold of 0.1% in SP and 18 in NSP), but similar when SP and NSP are compared. It is also obvious that most of the subpopulations existent in the bulk of NSP can also be found in the minor SP compartment, albeit at different proportions. For example in the IGROV1 cell line, the CD24^+^CD95^+^ (all other markers negative, blue) subset accounts for more than 50% of NSP but less than 25% of SP, where the CD24^+^CD95^+^HLA-ABC^+^ subset (green) predominates. Despite these differences between SP and NSP, no specific signature of SP subsets common to all cell lines could be detected. Interestingly though, the presence and proportion of the various subsets was stable over a period of several weeks (Suppl. Fig. 5). Importantly, when modifying the marker panel by exchanging two or three markers, additional subpopulations within the SP and the NSP became evident (Suppl. Fig. 6-12), although due to technical limitations in the number of fluorescence parameters, these subsets were not amenable to co-detection with the other subsets. Moreover, ALDH was also shown to contribute to SP and NSP heterogeneity by defining distinct subsets in both cell populations (data not shown). Finally in a proof-of-concept study in primary ovarian tumor tissue, we were able to detect a similar degree of intratumoral heterogeneity (Suppl. Fig. 4B), which corroborates our findings obtained from cell line models. Together, these data provide evidence for a high cellular complexity of both established ovarian cancer cell lines and freshly isolated primary ovarian tumor cells. Remarkably, even the stem-like SP compartment is composed of a plurality of distinct cell subsets.

## DISCUSSION

Our systematic screen with the ultimate goal to define a common CSC signature in ovarian cancer cell lines revealed that only populations expressing multidrug resistance proteins (SP cells) fulfilled the *bona fide* criteria of CSC. SP cells were detectable as minority populations in almost all cell lines and are characterized by enhanced clonogenic potential both *in vitro* and *in vivo*. Other markers evaluated were either not consistently expressed or did not define a population with CSC characteristics. Moreover, SP subsets were also detectable in primary ovarian tumor tissue. Therefore, our results extend previous studies (12-14) and suggest that the SP phenotype is a common and reliable marker for ovarian CSC.

Considering the significant subtype heterogeneity of ovarian cancer, it is quite remarkable that we found that a single marker can define stem cell populations in various ovarian tumor models. On the other hand, with the broad panel of cancer cell lines used, it was our declared goal to identify a consensus marker for ovarian CSC, hence to reduce the ambiguity that currently exists. Therefore, our study was specifically conceived to filter out, from cell lines with different histological origins, p53 status and tumor-biological resemblance [26, 27], a common signature for CSC and/or stem-like cancer cells. Accordingly, the identified marker (i.e., the SP phenotype) may be seen as the least common denominator of ovarian CSC in the investigated models rather than the ‘best’ CSC marker in each of the individual cell lines. Nonetheless, in view of established CSC signatures for other heterogeneous tumor entities (e.g., breast cancer) and the conservation of certain CSC markers throughout different tumor entities (e.g., CD133), we are confident that using a particular marker profile for definition of CSC from various ovarian tumor models is a reasonable approach.

Of note, we observed differential expression patterns of ABCG2 and ABCB1 among SP compartments of individual cell lines. Although such heterogeneity has been described previously [14], ABCG2- and ABCB1-expressing cancer cells have never been systematically compared on the functional level. We here show for the first time equivalence of ABCG2- and ABCB1-expressing ovarian cancer SP cells regarding stem cell properties such as clonogenicity, tumorigenicity, and asymmetric division. Thus, our results suggest similar associations of different drug transporters with stemness properties in ovarian cancer, and highlight the advantage of SP analysis to detect both ABCG2- and ABCB1-expressing cells, as well as other ABC transporters associated with CSC [28], compared to the determination of individual transporters. Nonetheless, heterogeneous ABCG2/ABCB1 expression implicates differential responsiveness to clinically relevant anticancer agents. For instance, ABCB1-expressing, but not ABCG2-positive, SP cells will be resistant to paclitaxel [3], an agent commonly used for ovarian cancer treatment.

We have used established ovarian carcinoma cell lines to initially address CSC and their heterogeneity assuming that these models provide a very robust basis for primary screening [29]. This is largely because cell lines are solely composed of cancer cells, which facilitates the detection and isolation of rare cell subsets. Conversely, primary tumor tissue also contains non-tumor cells and inherently underlies a high degree of inter-patient variation. In addition, it is difficult to enrich the scarce primary CSC-like cells from primary tumor tissue or malignant ascites to a cell count allowing phenotypic and functional characterization. Despite this, it is important to emphasize that we and others [11-13, 30] have detected SP cells also in tumor tissue and ascites of ovarian cancer patients, and that these primary SP cells exhibit stem cell characteristics as well [13]. In addition, SP cells seem to be enriched during chemotherapy *in vivo* [14].

It is important to mention that we, as most other investigators dealing with CSC characterization, found that functional stem cell features did not completely match the putative stem cell compartment [11, 31, 32], in our study defined by the SP phenotype. Specifically, our investigations demonstrated that NSP cells also exhibited a certain level of clonogenicity and tumorigenicity. Conversely, not all SP cells were clonogenic *in vitro*, and tumor formation by SP cells in immune-compromised animals required a relatively high number of cells. In this context, it is important to note that both clonogenicity and tumorigenicity may strongly depend on the specific model system used. For instance, grafting of tumor cells into more immune-compromised animals was shown to dramatically increase the frequency of tumorigenic cells [33]. Moreover, the injection site and whether or not the cells are injected in matrigel similarly impacts *in vivo* tumorigenicity [33, 34]. *In vitro*, CSC growth primarily depends on the culture conditions (e.g., growth factors, etc.) and the attachment surface [2]. In this study, we have chosen intra-peritoneal inoculation in aqueous solution to challenge the mice, based on the rationale that this site is orthotopic for ovarian cancer dissemination and that the tumor cells, due to random distribution throughout the abdominal cavity, are not deposited as an *in situ* tumor. Likewise, we chose a mesothelium-based co-culture model to investigate spheroid formation because mesothelial surfaces represent the most relevant microenvironment for disseminated ovarian cancer cells [35]. Interestingly, we found that the potential to form colonies after single cell sorting *in vitro* nicely reflected spheroid formation and *in vivo* tumorigenicity.

Our data tempt us to speculate that *bona fide* CSC, although enriched within the SP, only represent a specific subpopulation of SP cells, which may be defined by an additional marker constellation. In leukemia, for instance, stem cells are enriched in the CD34^+^ fraction and further enriched in the CD34^+^/CD38^−^ fraction, yet the ‘real’ leukemia-initiating cell is believed to be represented by an even smaller subpopulation of CD34^+^/CD38^−^ cells [36]. Thus, heterogeneity and plasticity within a stem cell-enriched population could also explain why ovarian cancer SP cells have increased but not 100 percent colony-forming potential.

To address this point in more detail, we further characterized SP compartments using a panel of markers not confined to classical CSC markers but also incorporating markers implicated in tumor progression/metastasis, EMT, and immune interaction. Intriguingly, these analyses revealed a tremendous heterogeneity not only of the bulk of NSP but also of the small SP compartment, ultimately preventing us from elaborating a more restricted ovarian CSC signature. This heterogeneity may at least in part explain why stem cell activity is not absolutely restricted to SP, and why plenty of different stem cell compartments have been reported in many solid tumor entities including ovarian cancer [2, 18].

Although tumor heterogeneity is well-known for its association with aggressive and/or advanced cancers [37, 38], the role of CSC heterogeneity remains elusive. Specifically, it remains unknown whether the various subsets within the SP compartment represent coexisting small CSC pools, or whether they reflect distinct stages of differentiation. Alternatively, the different subsets may arise spontaneously as a result of inherent plasticity [39], keeping them responsive to selective pressures. Of note, we found that the stability of SP heterogeneity was high, as the different cellular subsets were to be resolved repeatedly in independent experiments, each time exhibiting remarkably similar sizes. This indicated strict regulation of the subsets *via* defined molecular programs.

It is also widely accepted that tumor heterogeneity contributes to treatment failure by acting as a source of therapy-resistant cancer cells [40, 41]. Thus, it is plausible that heterogeneity of CSC also has implications for clinical drug resistance and immune escape. For instance, ALDH-positive SP cells may preferentially persist in patients due to detoxification of physiological or therapy-induced reactive aldehydes [42]. Likewise, HLA-class I low-expressing or CD95 (death receptor)-negative SP cells may be less susceptible to cytotoxic immune cell-mediated killing than their HLA-class I high-expressing or CD95-positive counterparts, respectively [43, 44]. On the other hand, a prominent tumorigenic and growth-promoting function of CD95 was recently discovered in ovarian and other cancers [45], and this could explain the consistently higher expression of CD95 in SP of essentially all cell lines analysed. These considerations suggest that even directed therapies with the ultimate goal to eradicate a specific CSC population should be based on a combination of several agents with non-overlapping modes of action.

To our knowledge, our study provides first systematic evidence for the exceedingly complex cellular composition of putative CSC compartments of solid tumors even in homogenous cell line models. In addition, our investigations on primary tumor cells showed a comparable degree of heterogeneity and disclosed putative stem cell compartments as well. Although we believe that the herein reported CSC heterogeneity is unprecedented, we appreciate that novel technologies allowing the simultaneous measurement of many more parameters at the single cell level will unravel an even higher number of subsets [46]. The big challenge, however, will be the functional characterization of these subsets. Certainly*, a priori* unselected** tumor formation assays in mice will not easily be feasible given the multitude of different populations to be tested and the restrictions discussed above. Surrogate *in vitro* tests like clonogenicity and spheroid formation will be necessary for initial screening approaches to identify promising cell populations for further *in vivo* testing. In addition, multidimensional flow cytometry together with other systems biology approaches will help to elucidate the genetic background, hierarchy and molecular pathways involved [46], opening new avenues for a personalized medicine in ovarian cancer.

In summary, based on systematic screening for stem cell characteristics in various cell lines and primary tumor tissue, we here provide strong evidence for the SP phenotype, conferred by several distinct ABC drug transporters, as marker of ovarian CSC. In the light of the tremendous heterogeneity we found both within an individual cell line and between cell lines, it is even more remarkable that SP was found in essentially all cell lines investigated. Based on this finding we have established a panel of cell lines with SP and NSP pairs, which can be used as valuable platform for ovarian CSC target identification, and for ovarian CSC-specific drug testing. Furthermore, our study provides novel insights into the heterogeneity of tumors that extends even to the small subpopulations of CSC. Thus, CSC may not represent the homogeneous population commonly assumed, but may be characterized by marked diversity. Further investigations are needed to confirm the heterogeneity of putative CSC also in other tumor entities as well as in the setting of SP-independent stem cell compartments. Provided that heterogeneous CSC fractions have specific functional correlates, CSC heterogeneity will have implications not only for tumor biology, but also for cancer immune escape and the modulation of the anticancer treatment response, necessitating the development of multi-targeted treatment strategies.

## METHODS

### Ovarian Cancer Cell Lines and Culture Conditions

A2780 cells were purchased from Sigma-Aldrich, Vienna, Austria. Caov-3 (HTB-75), MDAH-2774, OVCAR-3 (HTB-161) and SKOV-3 (HTB-77) cells were purchased from ATCC, Wesel, Germany. B2/92, B16/92, B17/92 and B74/93 cells [47] were a kind gift of Prof. C. Brumm, Mainz, Germany, and HOC-7 cells [48] were generously provided by Prof. C. Dittrich, Vienna, Austria. IGROV1 [49] and SKOV-6 [50] cells were kindly obtained from Prof. R. Brown, London, UK, and Prof. L. Old, New York City, NY, respectively, and the A2780 variant cell line A2780V [8] was generously provided by Prof. R. Zeillinger, Vienna, Austria. Cell lines were cultured in the appropriate medium (i.e., RPMI 1640, DMEM high glucose, MEM with Earle's Salts or McCoy's 5A; all from PAA, Pasching, Austria) supplemented with 10% (v/v) FBS (Biochrom, Berlin, Germany), 2 mM L-glutamine and 1x penicillin/streptomycin (both from Gibco, Lofer, Austria). Before exceeding 80% confluency, cells were split in accordance with standard cell culture procedures using a 1x conc. trypsin solution (Gibco) and subcultured at a density of 1×10^4^ cells/cm^2^.

### Flow Cytometry

Flow cytometric analyses and cell sorting were performed on a FACSAria I (BD Biosciences, Vienna, Austria). On some occasions, analyses were also performed on a FACSCanto II or, in case a violet excitation source was not required, on a FACSCalibur (both from BD Biosciences). Propidium iodide (eBioscience, Vienna, Austria) or 7-aminoactinomycin (BD Pharmingen, Vienna, Austria) staining was included in all staining protocols (except multicolor protocols) to discriminate viable from non-viable cells, and debris and doublets/aggregates were excluded based on FSC/SSC characteristics. Data were finally analyzed using FlowJo version 9.6 (Tree Star Software, Ashland, CA).

SP analysis and sorting was performed as previously described [51]. Briefly, cells were adjusted to a concentration of 1×10^6^ (analysis) or 5×10^6^ (cell sorting) cells/ml culture medium and stained with 10 μM Vybrant^®^ DyeCycle^TM^ Violet (DCV; Molecular Probes^®^, Eugene, OR) for 90 min at 37°C. For control purpose, aliquots were put aside and incubated in the presence of either 20 μM fumitremorgin C (FTC) or 50 μM verapamil (both inhibitors were from Sigma-Aldrich). After completion of incubation, cells were washed in >10x volume ice-cold PBS (PAA), chilled on ice and immediately analyzed or sorted by flow cytometry.

Detection of ALDH enzymatic activity was accomplished using the ALDEFLUOR^®^ test (STEMCELL Technologies^TM^, Grenoble, France) strictly according to manufacturer's instructions. In brief, cells were adjusted to a concentration of 1×10^6^ cells/ml ALDEFLUOR assay buffer and incubated for 30-45 min at 37°C in the presence of 1.5 μM of a fluorescent ALDH substrate. In control aliquots, the fluorescence reaction was quenched by the specific ALDH inhibitor diethylaminobenzaldehyde (DEAB). Cells were spun down and resuspended in appropriate volumes of ALDEFLUOR assay buffer. Cells were kept on ice and immediately analyzed or sorted by flow cytometry.

To determine the expression levels of various surface markers, respective monoclonal antibodies were used (see Suppl. Table 3 for full list of antibodies, and Suppl. Table 4 for multicolor staining protocols). Typically, 5×10^5^ (analysis) or 2×10^6^ (cell sorting) cells were stained in a volume of 100 μl. Staining was performed at pre-titrated concentrations for 30 min at 4°C in PBS supplemented with 1% (v/v) FBS. Excessive antibody was washed away in >10x volume PBS. In the case of biotin-conjugated primary antibodies, detection was accomplished by secondary labeling with fluorochrome-conjugated streptavidin (30 min at 4°C). Cells were kept on ice and analyzed or sorted by flow cytometry within one hour. Nonspecific binding was controlled using irrelevant antibodies and compensation was performed using compensation beads (BD Biosciences). Fluorescence-minus-one controls were included where indicated.

DCV/ALDH/surface marker co-stainings were performed in succession as described elsewhere [18].

### Single Cell Clonogenicity and Asymmetric Division

For assessment of the clonogenic potential of single cells, SP/NSP cells or ALDH^+^/ALDH^−^ cells were single cell-sorted into 96-well plates containing 150 μl fresh medium. Plates were incubated for two weeks and the number of wells harboring viable clones was counted using bright-field microscopy. Potential toxic effects of DCV were excluded in adequate control experiments (i.e., ^3^H-thymidine incorporation of DCV^dim^ vs. DCV^bright^ cells and single cell cloning of established, hence DCV-negative, SP and NSP fractions; data not shown).

The potential of SP or NSP cells to differentiate into the reciprocal cell population (i.e., to undergo an asymmetric division-like process) was assessed by performing SP detection of single cell-derived clones (either SP or NSP). To this end, clones were harvested two weeks after single cell sorting, expanded for one more week and then stained with DCV. The fraction of the reciprocal cell population present in the sample was used as measure for asymmetric division.

### Spheroid Formation on Primary Mesothelium

Primary mesothelial cells were isolated from a surgically resected piece of omentum using collagenase digestion after informed consent of the patient and approval by the local ethical review board. Isolated mesothelial cells, suspended in RPMI 1640, were seeded into 8-well chamber slides (Sigma-Aldrich) and grown to confluency to establish a monolayer. At the time of confluency, 1×10^3^ SP or NSP cells were added to the culture by flow sorting, followed by incubation for one week to allow spheroid formation. Quantification was done by counting the number of spheroids using an appropriate grid under the microscope. Before use in experiments, isolated mesothelial cells were characterized regarding cobblestone-like morphology and expression of established mesothelial markers (i.e., CD44^+^, CD325^+^, vimentin^+^, pan-cytokeratin^+^, CD324^−^) (data not shown).

### NOD/SCID Xenografts

Five to eight week old female NOD/SCID mice (NOD.CB17-*Prkdc^scid^*/NCrHsd) were purchased from Harlan Laboratories (Harlan, Udine, Italy). All experiments involving animals were approved by the local ethical review board and performed at the Central Laboratory Animal Facility of the Innsbruck Medical University in strict accordance with the regulations of the Austrian Animal Experiments Act. After initial dose finding experiments, 5×10^4^ tumor cells (either SP or NSP) were grafted into the mice's abdominal cavity by injecting 100 μl of cell suspensions. Tumor development was monitored twice a week and animals with severe tumor burden as evidenced by abdominal girth measurement were euthanized. Kaplan-Meier survival curves were used to analyze the data.

### Data Analysis and Statistics

Expression profiles as determined by flow cytometry were systematically analyzed using heatmap presentation. Heatmaps were generated using R version 3.0 (http://www.r-project.org/), and input data represented the MFI of the markers after normalization to irrelevant antibody controls and log-transformation. The cut-off for non-expressed antigens was set to 0.5, as this allowed maximum resolution. In multicolor experiments, analysis and presentation of distributions was performed using SPICE version 5.3, downloaded from http://exon.niaid.nih.gov/spice/ [52]. Input data represented a combination of Boolean gates, and the cut-off for *bona fide* subsets was set to 0.1% of total cells. Data were finally presented in the weighted category mode.

Unless otherwise stated, data are shown as mean ± SEM of at least three independent experiments. The statistical significance of the data was determined using two-tailed paired Student's t-test, with the exception of Kaplan-Meier survival data where the statistical significance was determined using log-rank test. P-values <0.05 were considered significant and marked ‘*’ (p-values <0.01 were marked ‘**’).

## SUPPLEMENTAL MATERIAL FIGURE AND TABLES




