# Supplementary material for: The side population of ovarian cancer cells defines a heterogeneous compartment exhibiting stem cell characteristics
Source: Oncotarget. 2014 Jun 1;5(16):7027–39. doi: 10.18632/oncotarget.2053 (PMC4196181; doi:10.18632/oncotarget.2053)
Supplement: Supplementary file 1 [file oncotarget-05-7027-s001.pdf]

Supplemental Table 1: Characteristics of human ovarian cancer cell lines used in this study.

Cell Line	Description	Supposed Histological Subtype	p53 Status	References
A2780	Derived from tumor tissue of an untreated patient	Endometrioid	wild-type	[1-4]
A2780V	Variant cell line of A2780	Same as A2780	unknown	[5]
B2/92	Derived from primary tumor tissue of an untreated patient	Poorly differentiated	unknown	[6]
B16/92	Derived from omental metastasis of an untreated patient	Poorly differentiated	unknown	[6]
B17/92	Derived from malignant ascites of an untreated patient	Serous	unknown	[6]
B74/93	Derived from pleural effusion of an untreated patient	Serous	unknown	[6]
Caov-3 (HTB-75)		High-grade serous	mutated	[1, 2, 7-9]
HOC-7	Well-differentiated stage III adenocarcinoma of the ovary, derived from ascites tumor	Low-grade serous	wild-type	[10-12]
IGROV1	Stage III ovarian cancer, derived from right ovary	Mixed/endometrioid	wild-type	[1, 13, 14]
MDAH-2774	Derived from malignant ascites	Endometrioid	mutated	[7, 9, 15-17]
OVCAR-3 (HTB-161)	Derived from tumor tissue of a patient refractory to cisplatin, derived from malignant ascites	High-grade serous	mutated	[1, 2, 4, 7, 18]
SKOV-3 (HTB-77)	Derived from malignant ascites	Atypical non-serous	mutated	[1, 8, 9, 19, 20]
SKOV-6		unknown	unknown	[21]

Note: There may be contradictory reports regarding the p53 status of particular cell lines. For instance, IGROV1 and SKOV-3 were also recently reported as mutant and wild-type for p53, respectively [2].

Supplemental Table 2: Screening of various ovarian cancer cell lines for commonly used CSC markers.

	A2780	A2780V	B2/92	B16/92	B17/92	IGROV1
**CD24**	2.22 ± 0.18	0.51 ± 0.14	98.7 ± 0.50	10.59 ± 0.38	2.29 ± 0.12	99.8 ± 0.15
**CD44**	—	—	3.92 ± 0.34	0.31 ± 0.01	—	24.5 ± 1.32
**CD90**	99.6 ± 0.40	99.6 ± 0.35	—	7.11 ± 0.88	0.51 ± 0.26	0.29 ± 0.07
**CD133**	0.30 ± 0.04	—	6.76 ± 1.06	0.44 ± 0.08	—	4.80 ± 0.67
**CD326**	0.51 ± 0.14	—	98.75 ± 0.55	0.32 ± 0.04	—	98.43 ± 1.17
**ALDH**	0.63 ± 0.06	0.08 ± 0.01	18.63 ± 0.85	4.10 ± 1.08	0.25 ± 0.09	1.21 ± 0.17
**SP**	0.62 ± 0.14	11.03 ± 0.86	0.77 ± 0.19	0.31 ± 0.09	0.08 ± 0.02	0.76 ± 0.25

Supplemental Table 3: List of antibodies used in this study.

Test Antibody	Alternate Names	Clone	Format	Supplier
CD24	BA-1, HSA	32D12, ML5	PE, PerCP/Cy5.5, APC	Miltenyi Biotec, BioLegend
CD44	H-CAM, Pgp-1	DB105	FITC	Miltenyi Biotec
CD49d	VLA-4α, Integrin α4	9F10	PE, APC-eFluor-780	BD Pharmingen™, eBioscience
CD80	B7-1, CD28LG1	L307.4	PE	BD Pharmingen™
CD86	B7-2, CD28LG2	2331 (FUN-1)	PE	BD Pharmingen™
CD90	Thy-1	5E10	APC	BioLegend
CD95	Fas, Apo-1	DX2	PE-Cy7, APC	eBioscience, BD Biosciences
CD106	VCAM-1	STA	PE	BioLegend
CD133	Prominin-1	293C3	APC	Miltenyi Biotec
CD140a	PDGFRα	αR1	PE	BD Pharmingen™
CD171	L1CAM	11a-9.3	Alexa Fluor 647	Prof. Dr. Peter Altevogt
CD178	FasL, CD95L	ALF-2.1A	FITC	Biomol
CD183	CXCR3	1C6/CXCR3	PE	BD Pharmingen™
CD184	CXCR4, Fusin	12G5	PE, PE-CF594	BD Pharmingen™, BD Horizon™
CD193	CCR3	5E8	Alexa Fluor 647	BD Pharmingen™
CD195	CCR5	3A9	PE	BD Pharmingen™
CD196	CCR6	11A9	PE	BD Pharmingen™
CD199	CCR9	112509	Alexa Fluor 647	BD Pharmingen™
CD200	OX-2	OX-108	PE	BioLegend
CD243	P-glycoprotein, ABCB1, MDR1	UIC2	PE, APC	eBioscience

CD317	BST2, Tetherin	26F8	Alexa Fluor 488	eBioscience
CD324	E-cadherin	67A4	FITC	BioLegend
CD325	N-cadherin	8C11	PE	BioLegend
CD326	EpCAM, ESA	HEA-125	FITC, PE-Cy7	Miltenyi Biotec, BioLegend
CD338	ABCG2, Bcrp1	5D3	APC, Biotin	BioLegend
HLA-ABC	MHC class I	G46-2.6	FITC	BD Pharmingen^{TM}
Streptavidin			BV421	BioLegend
**Control Antibody**	**Alternate Names**	**Clone**	**Format**	**Supplier**
CD4	T4, Leu-3a	SK3	FITC, PE, APC	BD Biosciences
CD21	C3DR, CR2	B-ly4	FITC	IQ Products
CD40	TNFRSF5	5C3	FITC	eBioscience
CD45	LCA, T200	TU116, MB4-6D6	PE, APC, APC-Cy7	BD Pharmingen^{TM}, Miltenyi Biotec, BioLegend
CD45RA		14.8	PE	BD Pharmingen^{TM}
CD62L	L-selectin	SK11	FITC	BD Biosciences

Supplemental Table 4. Multicolor staining protocols.

Staining 1	Staining 2	Staining 3	Staining 4	Staining 5	Staining 6
CD24	CD24	CD24	CD24	CD24	CD24
CD49d	CD49d	CD49d	CD44	CD44	CD44
CD90	CD95	CD95	CD49d	CD49d	CD49d
CD95	CD171	CD133	CD95	CD95	CD90
CD140a	CD184	CD184	CD140a	CD133	CD95
CD184	CD325	CD325	CD171	CD140a	CD184
HLA-ABC	HLA-ABC	HLA-ABC	CD184	CD184	CD325
SP	SP	SP	SP	SP	SP

SP……SP/NSP discrimination

Modifications:

<u>IGROV1 – Staining 2:</u> CD326 instead of HLA-ABC

<u>IGROV1 – Staining 5:</u> CD326 instead of CD44

References

1. Anglesio MS, Wiegand KC, Melnyk N, Chow C, Salamanca C, Prentice LM, Senz J, Yang W, Spillman MA, Cochrane DR, Shumansky K, Shah SP, Kalloger SE and Huntsman DG. Type-specific cell line models for type-specific ovarian cancer research. PloS one. 2013; 8(9):e72162.
2. Domcke S, Sinha R, Levine DA, Sander C and Schultz N. Evaluating cell lines as tumour models by comparison of genomic profiles. Nature communications. 2013; 4:2126.
3. Eva A, Robbins KC, Andersen PR, Srinivasan A, Tronick SR, Reddy EP, Ellmore NW, Galen AT, Lautenberger JA, Papas TS, Westin EH, Wong-Staal F, Gallo RC and Aaronson SA. Cellular genes analogous to retroviral onc genes are transcribed in human tumour cells. Nature. 1982; 295(5845):116-119.

4. Godwin AK, Meister A, O'Dwyer PJ, Huang CS, Hamilton TC and Anderson ME. High resistance to cisplatin in human ovarian cancer cell lines is associated with marked increase of glutathione synthesis. Proc Natl Acad Sci U S A. 1992; 89(7):3070-3074.
5. Zeimet AG, Reimer D, Sopper S, Boesch M, Martowicz A, Roessler J, Wiedemair AM, Rumpold H, Untergasser G, Concin N, Hofstetter G, Muller-Holzner E, Fiegl H, Marth C, Wolf D, Pesta M, et al. Ovarian cancer stem cells. Neoplasma. 2012; 59(6):747-755.
6. Marth C, Zeimet AG, Herold M, Brumm C, Windbichler G, Muller-Holzner E, Offner F, Feichtinger H, Zwierzina H and Daxenbichler G. Different effects of interferons, interleukin-1beta and tumor necrosis factor-alpha in normal (OSE) and malignant human ovarian epithelial cells. International journal of cancer Journal international du cancer. 1996; 67(6):826-830.
7. Kannan K, Coarfa C, Rajapakshe K, Hawkins SM, Matzuk MM, Milosavljevic A and Yen L. CDKN2D-WDFY2 Is a Cancer-Specific Fusion Gene Recurrent in High-Grade Serous Ovarian Carcinoma. PLoS genetics. 2014; 10(3):e1004216.
8. Fan S, Twu NF, Wang JA, Yuan RQ, Andres J, Goldberg ID and Rosen EM. Down-regulation of BRCA1 and BRCA2 in human ovarian cancer cells exposed to adriamycin and ultraviolet radiation. International journal of cancer Journal international du cancer. 1998; 77(4):600-609.
9. Lin J, Tang H, Jin X, Jia G and Hsieh JT. p53 regulates Stat3 phosphorylation and DNA binding activity in human prostate cancer cells expressing constitutively active Stat3. Oncogene. 2002; 21(19):3082-3088.
10. Buick RN, Pullano R and Trent JM. Comparative properties of five human ovarian adenocarcinoma cell lines. Cancer Res. 1985; 45(8):3668-3676.
11. King ER, Zu Z, Tsang YT, Deavers MT, Malpica A, Mok SC, Gershenson DM and Wong KK. The insulin-like growth factor 1 pathway is a potential therapeutic target for low-grade serous ovarian carcinoma. Gynecologic oncology. 2011; 123(1):13-18.
12. Mullany LK, Liu Z, King ER, Wong KK and Richards JS. Wild-type tumor repressor protein 53 (Trp53) promotes ovarian cancer cell survival. Endocrinology. 2012; 153(4):1638-1648.
13. Benard J, Da Silva J, De Blois MC, Boyer P, Duvillard P, Chiric E and Riou G. Characterization of a human ovarian adenocarcinoma line, IGROV1, in tissue culture and in nude mice. Cancer Res. 1985; 45(10):4970-4979.
14. O'Connor PM, Jackman J, Bae I, Myers TG, Fan S, Mutoh M, Scudiero DA, Monks A, Sausville EA, Weinstein JN, Friend S, Fornace AJ, Jr. and Kohn KW. Characterization of the p53 tumor suppressor pathway in cell lines of the National Cancer Institute anticancer drug screen and correlations with the growth-inhibitory potency of 123 anticancer agents. Cancer Res. 1997; 57(19):4285-4300.
15. Freedman RS, Pihl E, Kusyk C, Gallager HS and Rutledge F. Characterization of an ovarian carcinoma cell line. Cancer. 1978; 42(5):2352-2359.
16. Wang Y, Wu R, Cho KR, Shedden KA, Barder TJ and Lubman DM. Classification of cancer cell lines using an automated two-dimensional liquid mapping method with hierarchical clustering techniques. Molecular & cellular proteomics : MCP. 2006; 5(1):43-52.
17. Jacobberger JW, Sramkoski RM, Zhang D, Zumstein LA, Doerksen LD, Merritt JA, Wright SA and Shults KE. Bivariate analysis of the p53 pathway to evaluate Ad-p53 gene therapy efficacy. Cytometry. 1999; 38(5):201-213.
18. Hamilton TC, Young RC, McKoy WM, Grotzinger KR, Green JA, Chu EW, Whang-Peng J, Rogan AM, Green WR and Ozols RF. Characterization of a human ovarian carcinoma cell line (NIH:OVCAR-3) with androgen and estrogen receptors. Cancer Res. 1983; 43(11):5379-5389.

19. Pisanu ME, Ricci A, Paris L, Surrentino E, Liliac L, Bagnoli M, Canevari S, Mezzanzanica D, Podo F, Iorio E and Canese R. Monitoring response to cytostatic cisplatin in a HER2(+) ovary cancer model by MRI and in vitro and in vivo MR spectroscopy. Br J Cancer. 2014; 110(3):625-635.
20. Yaginuma Y and Westphal H. Abnormal structure and expression of the p53 gene in human ovarian carcinoma cell lines. Cancer Res. 1992; 52(15):4196-4199.
21. Wallen JW, Cate RL, Kiefer DM, Riemen MW, Martinez D, Hoffman RM, Donahoe PK, Von Hoff DD, Pepinsky B and Oliff A. Minimal antiproliferative effect of recombinant mullerian inhibiting substance on gynecological tumor cell lines and tumor explants. Cancer Res. 1989; 49(8):2005-2011.
