# Supplementary material for: The side population of ovarian cancer cells defines a heterogeneous compartment exhibiting stem cell characteristics
Source: Oncotarget. 2014 Jun 1;5(16):7027–39. doi: 10.18632/oncotarget.2053 (PMC4196181; doi:10.18632/oncotarget.2053)
Supplement: Supplementary file 2 [file oncotarget-05-7027-s002.pdf]

The side population of ovarian cancer cells defines a heterogeneous compartment exhibiting stem cell characteristics

Supplementary Material

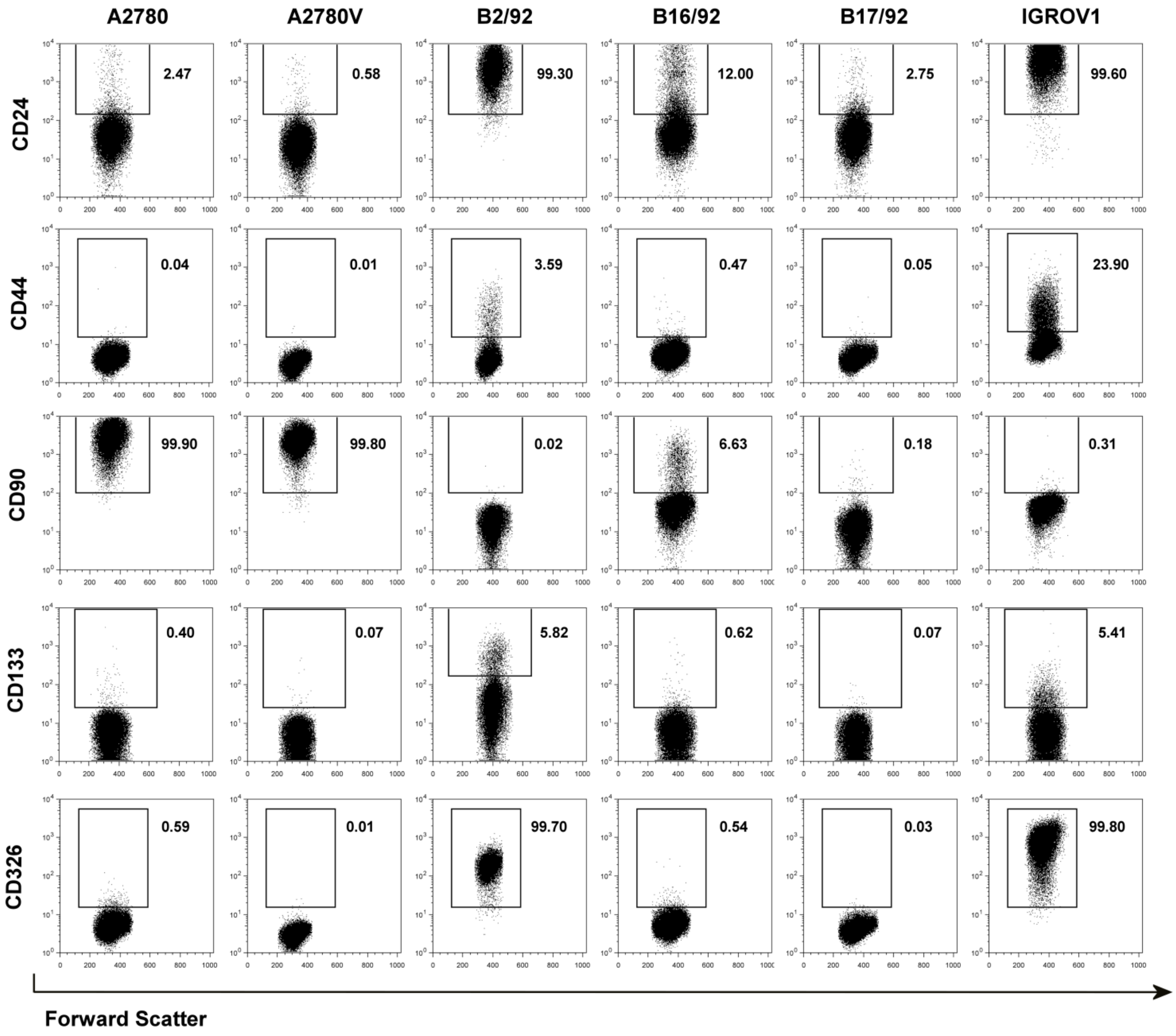

Supplemental Figure 1: Screening of various ovarian cancer cell lines for surface CSC marker subsets. Cell lines were stained using fluorochrome-conjugated monoclonal antibodies directed against surface CSC antigens. Subsets expressing CD24, CD44, CD90, CD133 and CD326 are indicated by rectangular gates, and the percentage of cells within these gates is given. Data are representative examples of three independent experiments. Nonspecific binding was controlled using control antibodies. CSC, cancer stem cell.

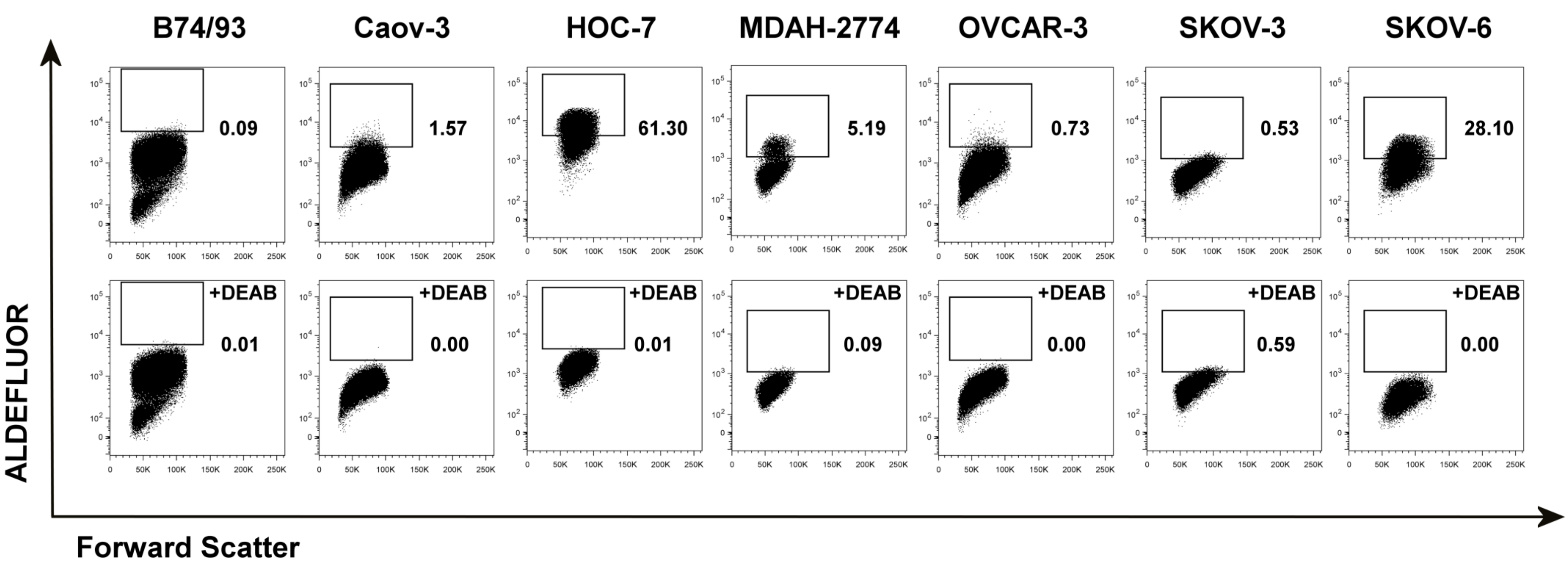

Supplemental Figure 2: Screening of additional ovarian cancer cell lines for ALDH+ subsets. Cell lines were stained for ALDH enzymatic activity and analyzed by flow cytometry. ALDH+ subsets are indicated by rectangular gates, and the percentage of cells within these gates is given (upper row). Corresponding DEAB inhibition controls are shown in the lower row. Data are representative examples of two independent experiments. ALDH, aldehyde dehydrogenase; DEAB, diethylaminobenzaldehyde.

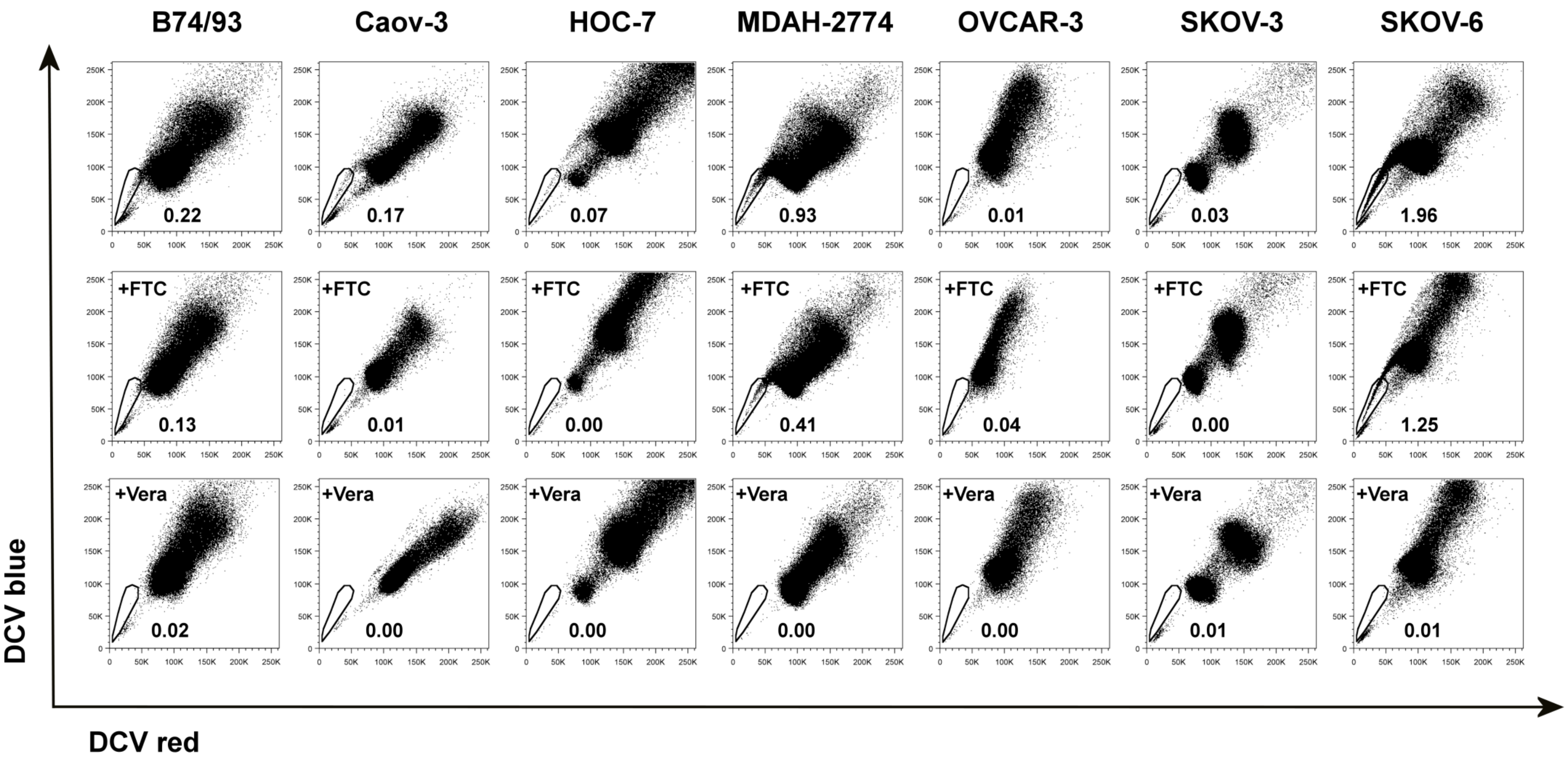

Supplemental Figure 3: Screening of additional ovarian cancer cell lines for SP subsets. Cell lines were stained using DCV and analyzed by flow cytometry. SP subsets are indicated by polygonal gates, and the percentage of cells within these gates is given (first row). Corresponding FTC (second row) and verapamil (Vera; third row) inhibition controls are shown. Data are representative examples of two independent experiments. SP, side population; DCV, Vybrant® DyeCycle™ Violet; FTC, fumitremorgin C.

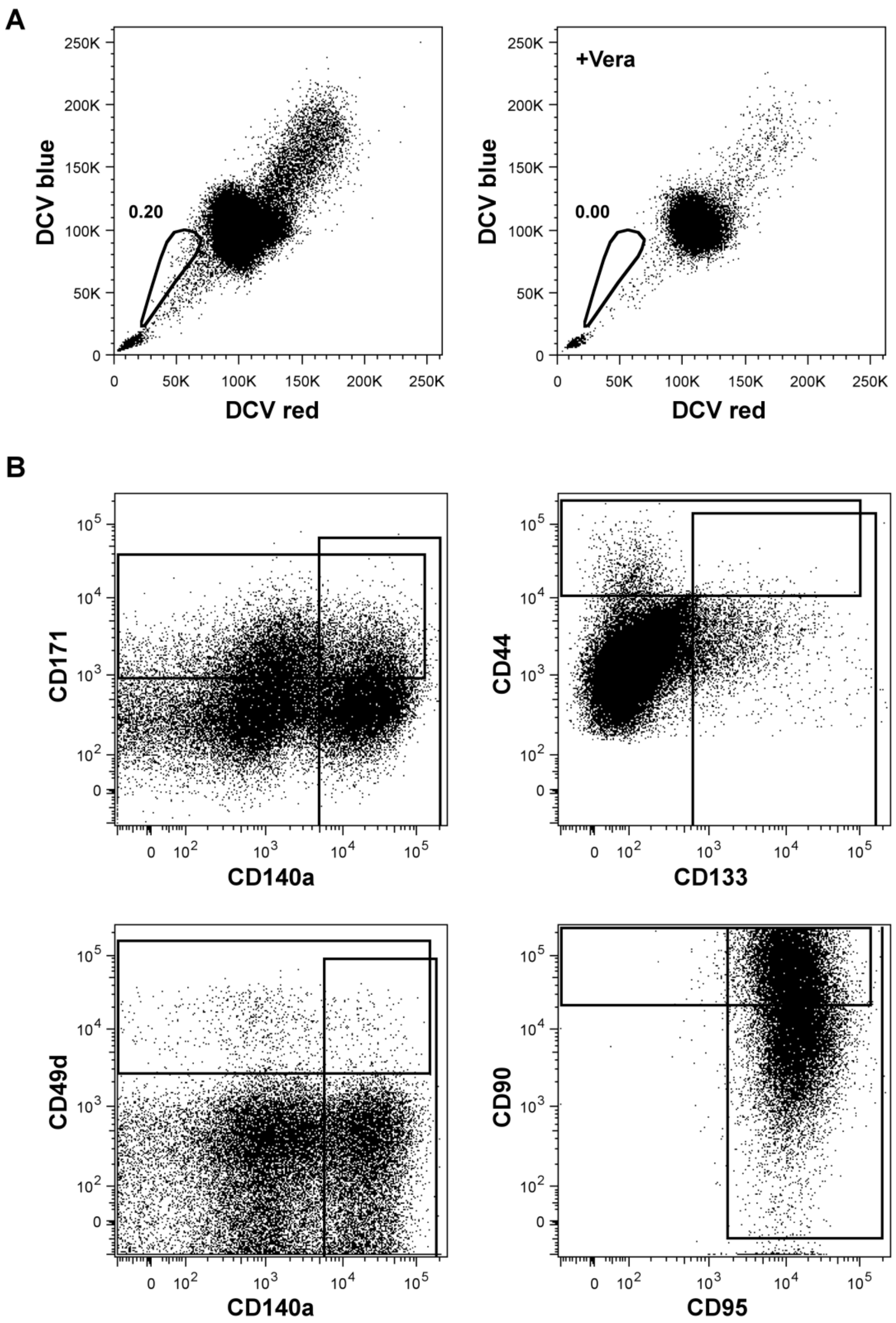
A
+Vera
0.20
0.00
DCV blue
DCV red
250K
200K
150K
100K
50K
0
B
CD171
CD140a
CD44
CD133
CD49d
CD90
CD95

Supplemental Figure 4: SP detection and analysis of heterogeneity in primary ovarian tumor tissue. Freshly isolated ovarian tumor tissue was dissociated into single cell suspensions using the gentleMACS^{TM} dissociator technology (Miltenyi Biotec, Bergisch Gladbach, Germany) and collagenase/DNAse digestion. Recovered cells were washed and filtered through 40 µm cell strainers. Thereafter, cells were stained for various markers and processed for flow cytometry. Staining protocols included discrimination markers for dead cells (7-aminoactinomycin), leukocytes (CD45) and epithelial cells (CD326), with the depicted plots being gated on the CD326^{pos}/CD45^{neg}/7-aminoactinomycin^{neg} tumor cell fraction. (A) DCV-based SP detection in primary ovarian tumor cells. The SP subset is indicated by a polygonal gate, and the percentage of cells within this gate is given (left panel). A corresponding verapamil (Vera) inhibition is shown as a control (right panel). (B) Analysis of heterogeneity in primary ovarian tumor cells using CD44, CD49d, CD90, CD95, CD133, CD140a and CD171 staining. The positive subsets for each of the individual markers are indicated by rectangular gates. As for cell lines, most markers show a biphasic expression pattern consistent with cellular heterogeneity. SP, side population; DCV, Vybrant® DyeCycle^{TM} Violet.

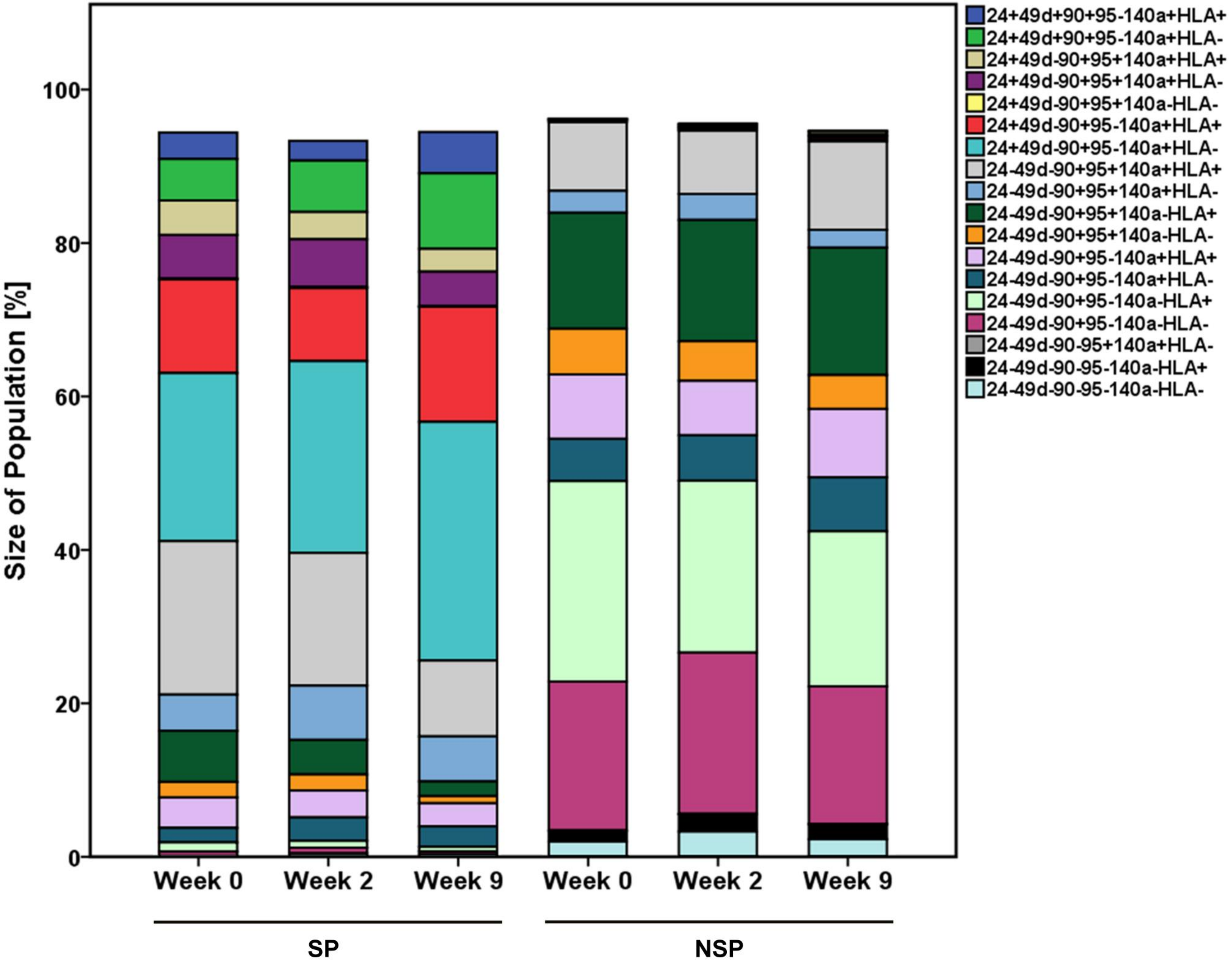

24+49d+90+95-140a+HLA+
24+49d+90+95-140a+HLA-
24+49d-90+95+140a+HLA+
24+49d-90+95+140a+HLA-
24+49d-90+95+140a-HLA-
24+49d-90+95-140a+HLA+
24+49d-90+95-140a+HLA-
24-49d-90+95+140a+HLA+
24-49d-90+95+140a+HLA-
24-49d-90+95+140a-HLA+
24-49d-90+95+140a-HLA-
24-49d-90+95-140a+HLA+
24-49d-90+95-140a+HLA-
24-49d-90+95-140a-HLA+
24-49d-90+95-140a-HLA-
24-49d-90-95+140a+HLA-
24-49d-90-95-140a-HLA+
24-49d-90-95-140a-HLA-
Size of Population [%]
0
20
40
60
80
100
Week 0
Week 2
Week 9
Week 0
Week 2
Week 9
SP
NSP

Supplemental Figure 5: Subset distribution of SP and NSP fractions over time. The composition of purified SP and NSP fractions was determined by multicolor flow cytometry at weeks 0, 2 and 9 (cell line: A2780). The stability of the principal subsets resolvable by the marker combination CD24, CD49d, CD90, CD95, CD140a and HLA-ABC, which cumulatively account for >90 of total cells both within SP and NSP, is depicted. Similar results were obtained with the cell line IGROV1 (data not shown). SP, side population; NSP, non-SP.

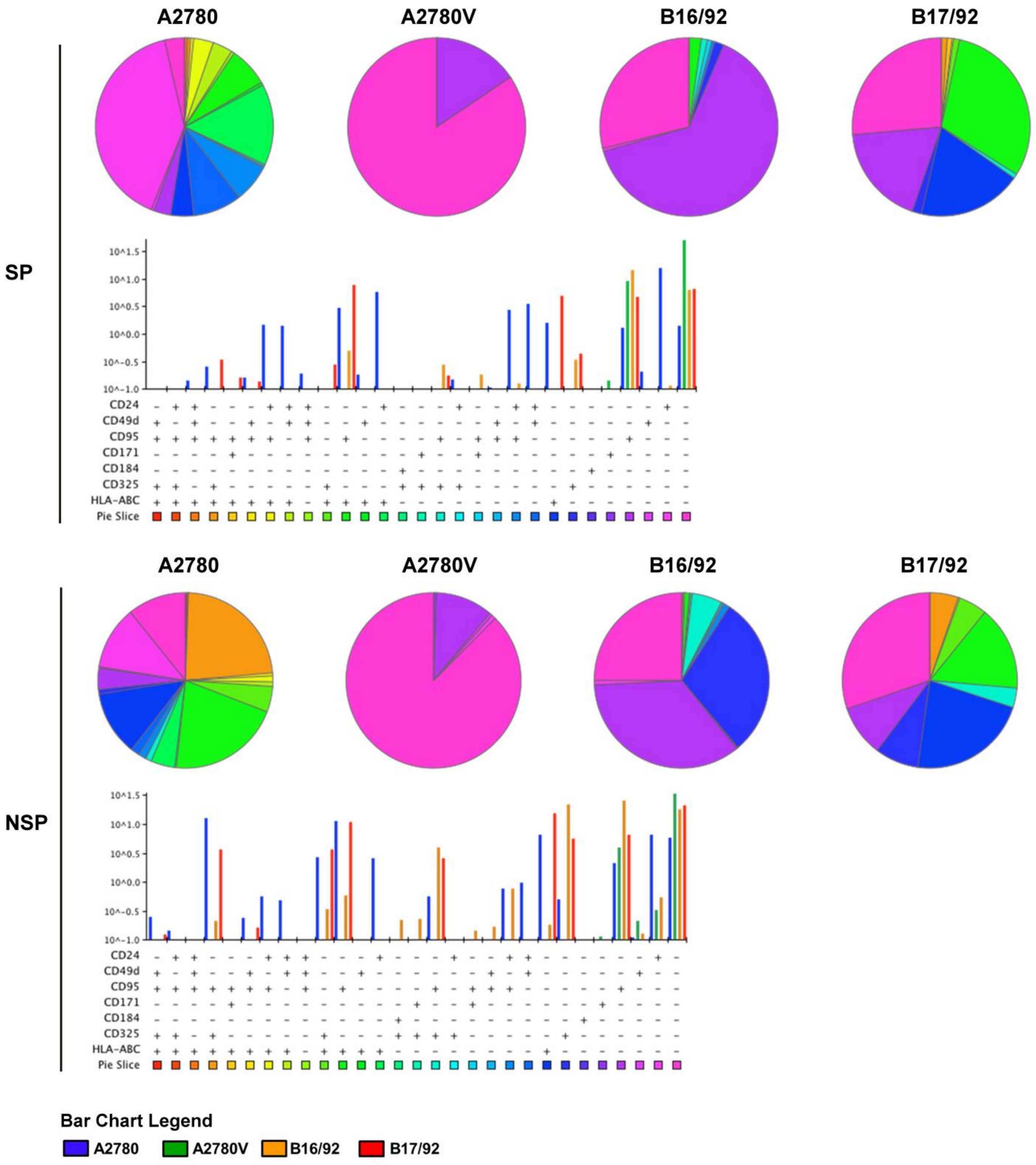

A2780
A2780V
B16/92
B17/92
SP
NSP
10^1.5
10^1.0
10^0.5
10^0.0
10^-0.5
10^-1.0
CD24
CD49d
CD95
CD171
CD184
CD325
HLA-ABC
Pie Slice
Bar Chart Legend
A2780
A2780V
B16/92
B17/92

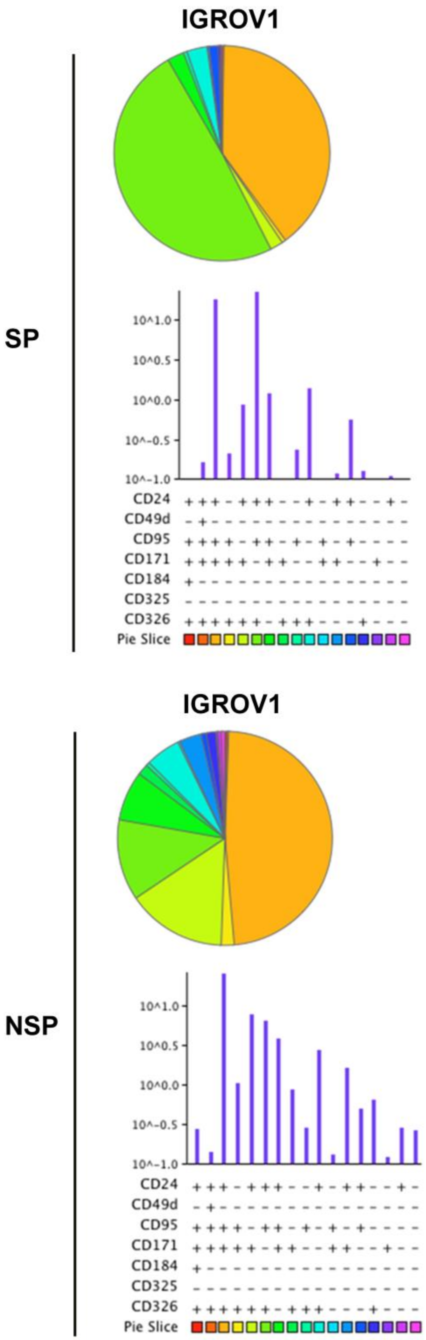
IGROV1
SP
10^1.0
10^0.5
10^0.0
10^-0.5
10^-1.0
CD24
CD49d
CD95
CD171
CD184
CD325
CD326
Pie Slice
IGROV1
NSP
10^1.0
10^0.5
10^0.0
10^-0.5
10^-1.0
CD24
CD49d
CD95
CD171
CD184
CD325
CD326
Pie Slice

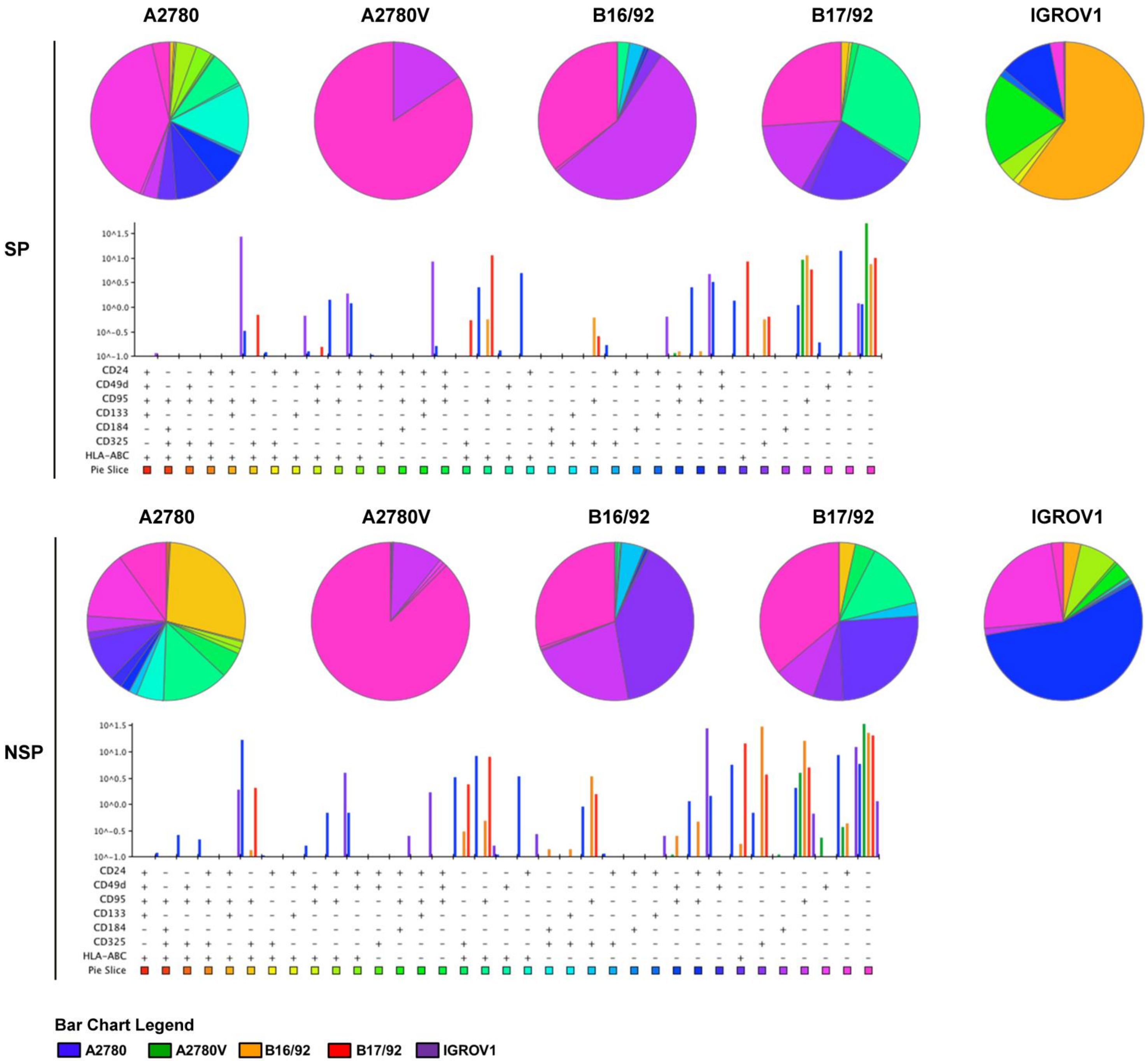
A2780
A2780V
B16/92
B17/92
IGROV1
SP
10^1.5
10^1.0
10^0.5
10^0.0
10^-0.5
10^-1.0
CD24
CD49d
CD95
CD133
CD184
CD325
HLA-ABC
Pie Slice
NSP
Bar Chart Legend
A2780
A2780V
B16/92
B17/92
IGROV1

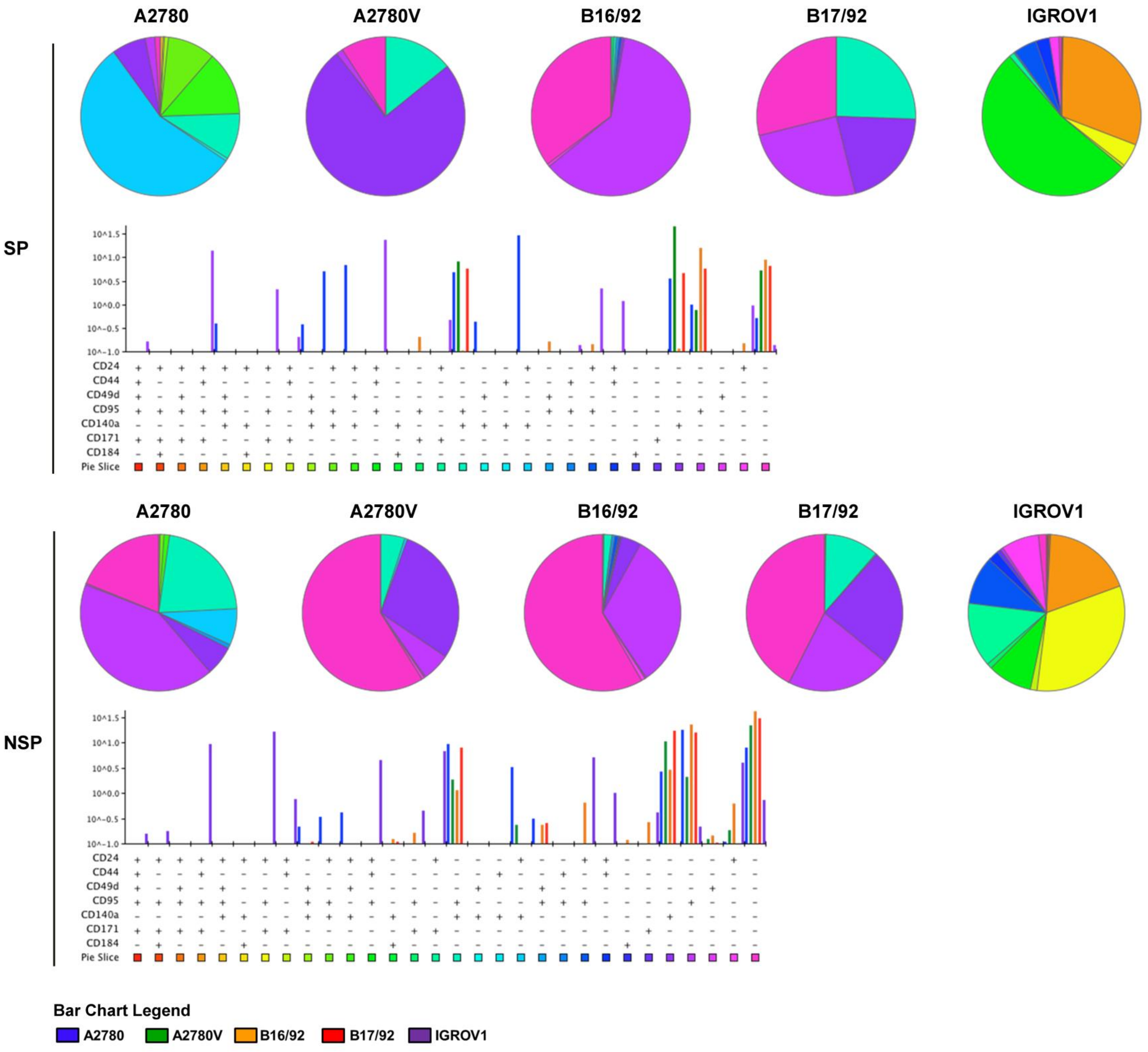

SP
A2780
A2780V
B16/92
B17/92
IGROV1
10^-1.0
10^-0.5
10^0.0
10^0.5
10^1.0
10^1.5
CD24
CD44
CD49d
CD95
CD140a
CD171
CD184
Pie Slice
NSP
A2780
A2780V
B16/92
B17/92
IGROV1
10^-1.0
10^-0.5
10^0.0
10^0.5
10^1.0
10^1.5
CD24
CD44
CD49d
CD95
CD140a
CD171
CD184
Pie Slice
Bar Chart Legend
A2780
A2780V
B16/92
B17/92
IGROV1

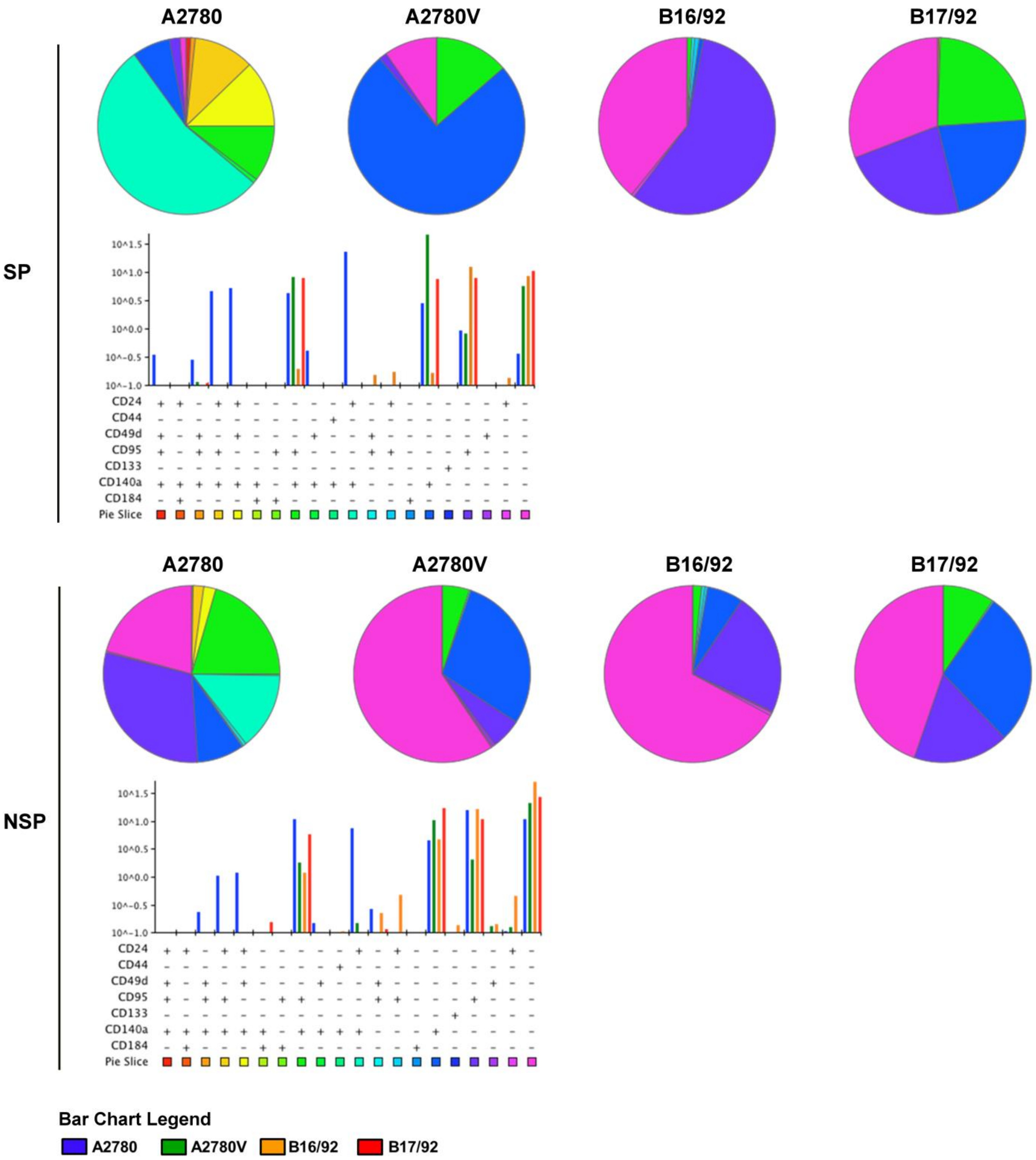
A2780
A2780V
B16/92
B17/92
SP
10^1.5
10^1.0
10^0.5
10^0.0
10^-0.5
10^-1.0
CD24
CD44
CD49d
CD95
CD133
CD140a
CD184
Pie Slice
A2780
A2780V
B16/92
B17/92
NSP
10^1.5
10^1.0
10^0.5
10^0.0
10^-0.5
10^-1.0
CD24
CD44
CD49d
CD95
CD133
CD140a
CD184
Pie Slice
Bar Chart Legend
A2780
A2780V
B16/92
B17/92

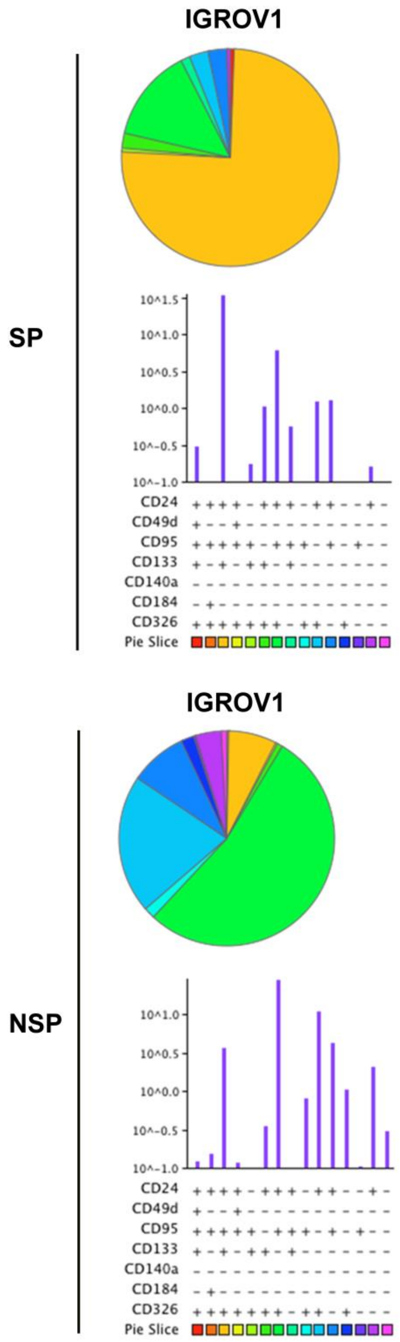
IGROV1
SP
10^1.5
10^1.0
10^0.5
10^0.0
10^-0.5
10^-1.0
CD24
CD49d
CD95
CD133
CD140a
CD184
CD326
Pie Slice
IGROV1
NSP
10^1.0
10^0.5
10^0.0
10^-0.5
10^-1.0
CD24
CD49d
CD95
CD133
CD140a
CD184
CD326
Pie Slice

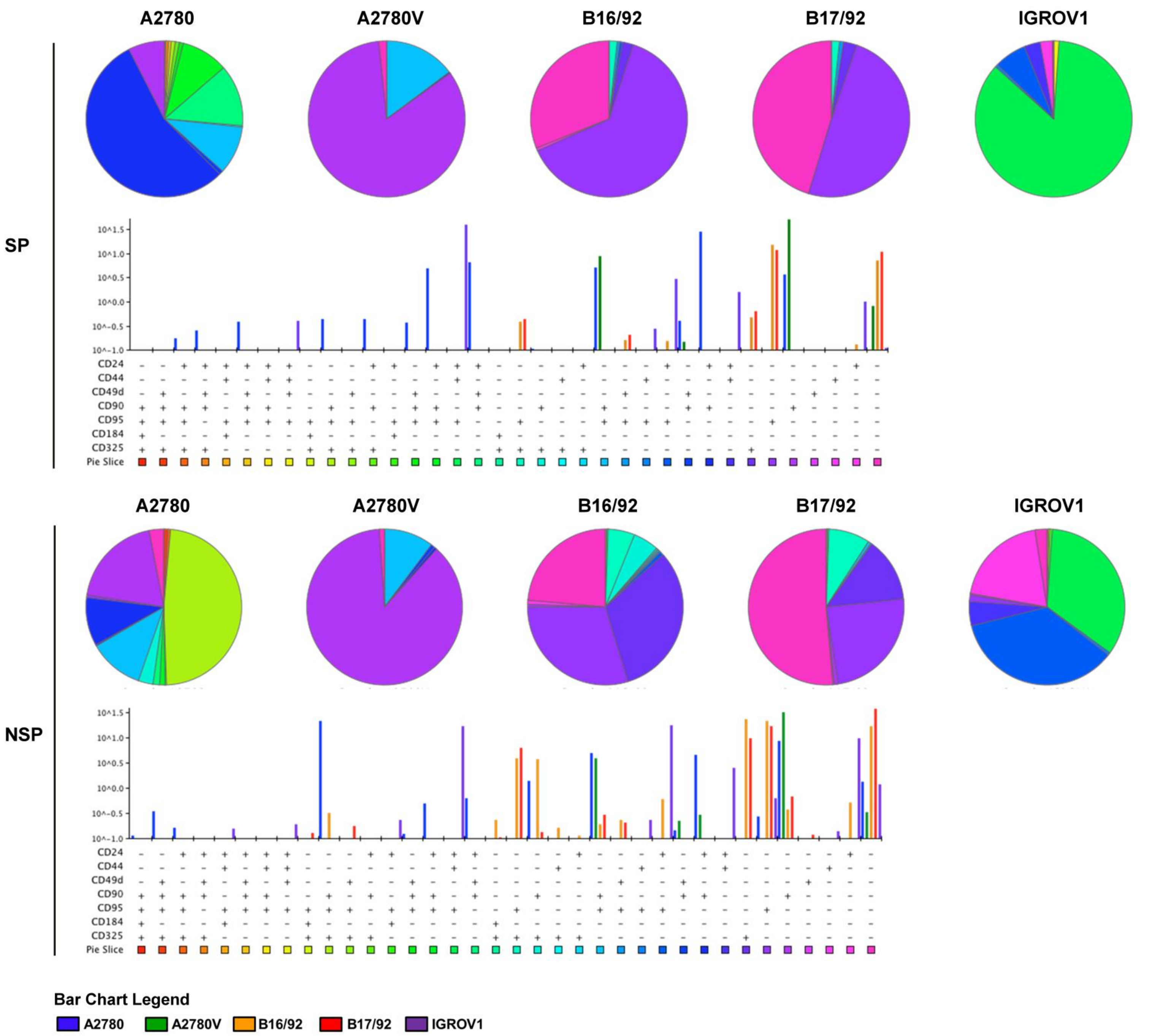

A2780
A2780V
B16/92
B17/92
IGROV1
SP
10^1.5
10^1.0
10^0.5
10^0.0
10^-0.5
10^-1.0
CD24
CD44
CD49d
CD90
CD95
CD184
CD325
Pie Slice
A2780
A2780V
B16/92
B17/92
IGROV1
NSP
10^1.5
10^1.0
10^0.5
10^0.0
10^-0.5
10^-1.0
CD24
CD44
CD49d
CD90
CD95
CD184
CD325
Pie Slice
Bar Chart Legend
A2780
A2780V
B16/92
B17/92
IGROV1

Supplemental Figures 6-12: SPICE analysis of additional ovarian cancer heterogeneity. Defined mixtures of corresponding SP and NSP fractions were stained for eight markers (stainings 2-6 of Suppl. Table 4) and subsequently analyzed by multicolor flow cytometry. Positive subsets were defined for each marker. These gates were combined by Boolean operations to obtain proportions of cells within all possible combinations, which were then imported into SPICE for final data analysis. SPICE analysis of ovarian cancer heterogeneity after class-division into SP (upper panel) and NSP (lower panel) fractions. Subset distributions are presented in the weighted category mode, and only subsets fulfilling the cut-off requirement of ≥0.1% are displayed. Data are representative examples of at least two independent experiments. SP, side population; NSP, non-SP.
